# Is It Safe to Use Arterial Grafts in Patients with Acute Myocardial Infarction? Short-Mid-Term Propensity Analysis

**DOI:** 10.21470/1678-9741-2023-0384

**Published:** 2024-10-18

**Authors:** Leonardo Lacava, Gabrielle Barbosa Borgomoni, Leticia de Mendonça Lopes, Leonardo Passaglia de Freitas, Fabiane Leticia Freitas, Luís Roberto Palma Dallan, Luiz Augusto Ferreira Lisboa, José Carlos Nicolau, Fabio B. Jatene, Omar Asdrúbal Vilca Mejia, Grupo de Estudos REPLICCAR (REPLICCAR Study Group)

**Affiliations:** 1 Department of Cardiovascular Surgery, Hospital Regional São Paulo (HRSP), Xanxerê, Santa Catarina, Brazil; 2 Department of Cardiovascular Surgery, Instituto do Coração, Hospital das Clínicas, Faculdade de Medicina, Universidade de São Paulo, São Paulo, São Paulo, Brazil; 3 Department of Cardiovascular Surgery, Hospital Paulistano, São Paulo, São Paulo, Brazil

**Keywords:** Coronary Artery Bypass, Myocardial Infarctation, Mammary Arteries, Reoperation, Propensity Score.

## Abstract

**Introduction:**

The use of multiple arterial grafts (MAGs) has an impact on patient survival; however, preference for its use in the acute phase of myocardial infarction (AMI) has not yet been established. This study aimed to compare the short-mid-term clinical results of AMI patients undergoing coronary artery bypass grafting (CABG) with a single arterial graft (SAG) vs. MAGs.

**Methods:**

This is a cross-sectional cohort study of 4,053 patients from the Registro Paulista de Cirurgia Cardiovascular II (REPLICCAR II). CABG in the AMI was considered when performed between one and seven days after diagnosis (n=238). Thirty-five patients underwent surgery with ≥ 2 arterial grafts (MAG group), population adjustment in SAG group was performed using the propensity score matching (PSM). Clinical follow-up was performed by telephone to assess need for readmission, new AMI, reoperation, and death.

**Results:**

After PSM, 70 patients were evaluated. During hospitalization, a significant statistical difference was observed in the surgery duration: the MAG group had a median of 4.78 hours while the SAG group had 4.11 hours (P=0.040). Within the MAG group, there was a predominance use of bilateral internal thoracic artery (62.86%), followed by radial graft associated with the use of left internal thoracic artery (28.57%) and the combination of the three grafts (8.57%). There were no significant differences between the groups in terms of outcomes up to 30 days after CABG or up to five years after CABG.

**Conclusion:**

In REPLICCAR II, usage of MAGs in the AMI was not associated with clinical worsening of patients until the mid-term follow-up.

## INTRODUCTION

Coronary artery bypass grafting (CABG) has a significant impact on survival of patients with coronary artery disease. The use of multiple arterial grafts (MAGs) plays an important role, due to its advantage related to long-term graft patency. Thus, guidelines recommend the use of a second arterial graft, mainly for the second best coronary artery^[1^-^4]^. However, this choice is not yet recommended in the acute phase of myocardial infarction (AMI) mainly due to the lack of evidence.

Database studies have already shown that the use of bilateral internal thoracic artery (BITA) is superior to single internal thoracic artery graft in long-term follow-up, even when revascularization is incomplete^[1^-^4]^. However, the time taken to harvest arterial grafts and the technical difficulty, with a consequent increase in surgical time, make most surgeons opt for the use of venous grafts in the AMI^[3,5,6]^. In addition, glycemic decompensation, presence of kidney failure, and the use of antiplatelet agents, common in this phase, have an influence on decision-making^[6]^.

Believing that by using more venous grafts we are reducing surgical time may be a false impression since recent studies showed a decrease in surgery time and wound infection rates besides an excellent long-term patency when a radial graft is associated with the internal thoracic artery^[7]^. Although the graft choice in CABG should always be individualized, we still have no evidence whether this can be modified by a situation of surgical urgency. In this scenario, groups with high-volume CABG surgeons have higher rates of use of MAGs with better results than groups with low-volume CABG surgeons^[8]^. Thus, perhaps we are depriving patients of these benefits due to the lack of evidence in the AMI.

The objective of this study was to compare the mid-term clinical results of patients operated on in the AMI with MAGs *vs.* single arterial graft (SAG) based on data registered in the Registro Paulista de Cirurgia Cardiovascular II (REPLICCAR II).

## METHODS

This is a retrospective cohort analysis of the REPLICCAR II database, a prospective, observational and multicenter registry with data from patients undergoing CABG consecutively in five hospitals in the state of São Paulo (Brazil) between July 2017 and June 2019 (n=4,053). REPLICCAR II followed the same variables and definitions of the Society of Thoracic Surgeons Adult Cardiac Surgery Database (STS ACSD) version 2.9 data collection system, through the partnership with the Harvard School of Public Health. All perioperative collection variables were performed online on a dedicated platform built on REDCap. Mid-term follow-up was carried out in a structured manner using a form filled out by telephone between October and December 2022. The AMI was considered 1-7 days of the diagnosis before the surgery, which followed the Fourth Universal Definition of Myocardial Infarction (2018)^[9]^.

This study included patients with AMI who underwent primary isolated CABG, while the exclusion criteria included elective procedures, combined surgeries, and reoperations. Definitions of the clinical status of the patient to guide revascularization follow the guidelines of the American societies of coronary revascularization of 2021^[10]^. Thirty-six patients were operated on in the AMI using MAGs, one patient who died in the operating room was excluded because the purpose of this analysis was to evaluate the mid-term clinical follow-up of patients. Therefore, the final sample resulted in 35 patients operated using MAGs. Using the propensity score matching (PSM), these patients were compared with other 35 patients with a similar baseline profile who were operated on under the same clinical conditions, however, who received a SAG in the procedure. Therefore, the immediate and mid-term clinical results of patients operated on with ≥ 2 arterial grafts (MAG group) *vs.* patients with one arterial graft (SAG group) in the AMI were compared in relation to need for readmission, new AMI, reoperation, and death from all causes.

**Fig. 1:**
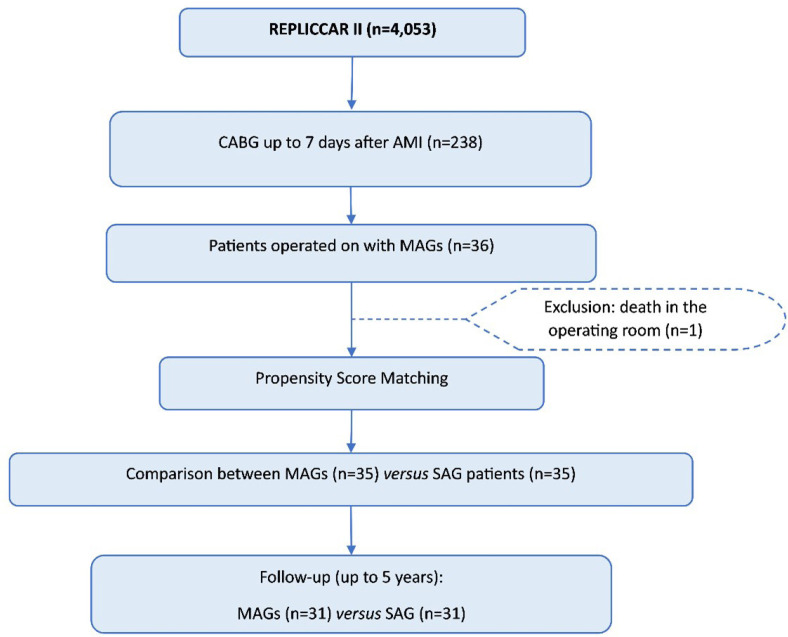
Methodology flowchart. AMI=acute phase myocardial infarction; CABG=coronary artery bypass grafting; MAGs=multiple arterial grafts; SAG=single arterial graft; REPLICCAR II=Registro Paulista de Cirurgia Cardiovascular II.

The methodology flowchart is shown in Figure 1.

### Data Collection

Clinical outcome variables included in the five-year follow-up survey form were: reinfarction, rehospitalization, reoperation, and death from all causes. All variable definitions followed the STS ACSD version 2.9 criteria^[11]^. Likewise, stroke, acute kidney failure, prolonged intubation, deep sternal wound infection, reoperation, and operative mortality were also compared in both groups.

### Ethics and Consent

This is a subanalysis of the REPLICCAR II project, approved by the Research Ethics Committee (Comissão de Ética para Análise de Projetos de Pesquisa [CAPPesq]) of the Hospital das Clínicas of the Universidade de São Paulo, opinion number 5,603,742, under CAAE registration number 66919417.6.1001.0068 and SDC number 4506/17/006. Informed consent was waived in the initial data collection due to the research design methodology applied to the project. In the follow-up analysis, the informed consent was obtained by telephone call from all study participants by registration number 5603742. The REPLICCAR Registry and The Statewide Quality Improvement Initiative ID in clinical trials is NCT05363696.

### Statistical Analysis

R software version 4.0.2 was used to carry out all the analyzes conducted in this study.

In the descriptive analysis, continuous variables were expressed as mean, median, standard deviation, and quartiles (25th/75th percentiles), while categorical variables were expressed in terms of frequencies and percentages. Due to missing data, percentages were calculated using the number of responses obtained by variables instead of the total number of patients.

PSM was used to pair the groups using the GenMatch function, available in the MatchIt package of R software, and its quality was verified by using the standardized mean difference (SMD) method (Table 1). The following variables were used to match the groups: sex, age, diabetes management, creatinine level, left ventricular ejection fraction, and need for intravenous nitrates (in the last 24 hours before surgery).

**Table 1:** Standardized mean difference (SMD) before and after propensity score matching (PSM).

PSM variable	SMD before PSM	SMD after PSM
Sex	0.144	0.085
Age	0.618	0.224
Diabetes management	0.536	0.144
Creatinine level	0.143	0.042
Left ventricular ejection fraction	-0.269	-0.078
Need for intravenous nitrates (in the last 24 hours before surgery)	0.247	0.085

SMD interpretation: 0 - 0.2, almost no difference; 0.2 - 0.5, little difference; 0.5 - 0.8, medium difference; 0.8 - 1, big difference

For the comparison of continuous variables from the two groups, *t*-test was used for normally distributed variables (Anderson-Darling test) and non-parametric tests were used for the other variables. Mann-Whitney test was used for homogeneous variables, and the Brunner-Munzel test was used for heterogeneous variables. Regarding categorical variables, Fisher's exact test or chi-square test was used. The significance level adopted in the tests was 0.05. Two-tailed hypotheses were considered. Furthermore, the constructed confidence intervals have a 95% confidence level.

## RESULTS

In the AMI, 238 patients underwent CABG; of these, 35 patients received MAG (15%) in REPLICCAR II. After pairing with 35 patients from the SAG group, the groups did not present statistically significant differences between the preoperative variables (Table 2).

**Table 2:** Propensity score matching preoperative variables.

Variable	SAGs		MAGs		*P*-value
	(n=35)		(n=35)		
	n	%	n	%	
Age, years, mean ± SD	60.17	± 7.19	58.49	± 8.06	0.510
Sex					1
Female	4	11.43%	5	14.29%	
Male	31	88.57%	30	85.71%	
LVEF (%), mean ± SD	52.82	± 10.31	53.78	± 14.29	0.421
Hematocrit (%), mean ± SD	40.53	± 4.84	40.27	± 4.56	0.530
Hemoglobin (mg/dL), mean ± SD	13.72	± 1.68	13.76	± 1.56	0.881
Glycosylated hemoglobin (%), mean ± SD	7.44	± 2.16	7.04	± 2.10	0.502
Blood glucose (mg/dL), mean ± SD	135.27	± 55.5	157.04	± 73.21	0.446
Creatinine (mg/dL)	1.10	± 0.26	1.09	± 0.26	0.874
Body mass index, mean ± SD	28.20	± 4.54	27.42	± 3.34	0.653
STS mortality score (%), mean ± SD	0.92	± 0.52	0.82	± 0.54	0.275
CCS angina classification					0.3
I	18	52.94%	15	46.88%	
II	10	29.41%	5	15.62%	
III	2	5.88%	4	12.50%	
IV	4	11.76%	8	25%	
NYHA classification					0.305
I	28	82.35%	29	90.62%	
II	4	11.76%	2	6.25%	
III	0	0%	1	3.12%	
IV	2	5.88%	0	0%	
Cancer in the last 5 years	1	2.86%	2	5.71%	1
Number of vessels affected					1
Two	7	20.59%	6	17.65%	
Three	27	79.41%	28	82.35%	
Smoker					0.801
Never	13	37.14%	12	36.36%	
Every day	6	17.14%	5	15.15%	
Smoker, frequency unknown	3	8.57%	2	6.06%	
Former smoker	11	31.43%	9	27.27%	
Diabetes mellitus	16	45.71%	17	48.57%	1
Diabetes treatment					1
Uncontrolled	2	12.50%	2	11.76%	
Oral hypoglycemic agents	8	50%	8	47.06%	
Insulin	4	25%	4	23.53%	
Unknown	2	12.50%	3	17.65%	
Systemic arterial hypertension	25	71.43%	28	80%	0.578
Dyslipidemia	16	45.71%	18	51.43%	0.811
Syncope	3	8.57%	1	2.86%	0.614
Peripheral artery disease	0	0.00%	3	8.57%	0.238
Intravenous nitrates in the last 24 hours before surgery	4	11.43%	5	14.29%	1
Intravenous inotropic within 48 hours before the surgery	3	8.57%	0	0%	0,238
Clinical symptoms at the time of surgery					0.885
No symptoms	24	68.57%	26	74.29%	
Unstable angina	1	2.86%	0	0%	
Non-STEMI	7	20%	6	17.14%	
STEMI	1	2.86%	1	2.86%	
Urgency status cause					0.206
AMI	26	89.66%	29	100%	
Ongoing cardiac ischemia	2	6.9%	0	0%	
Anatomical reason	1	3.45%	0	0%	

AMI=acute phase of myocardial infarction; CCS=Canadian Cardiovascular Society; LVEF=left ventricular ejection fraction; MAG=multiple arterial grafts; NYHA=New York Heart Association; SAG=single arterial graft; SD=standard deviation; STEMI=ST-elevation myocardial infarction; STS=Society of Thoracic Surgeons

Regarding the intraoperative period (Table 3), there was a significant statistical difference in surgery duration (period between surgical incision and referral to the intensive care unit), where the MAG group had a median of 4.78 hours while the SAG group had 4.11 hours (*P*=0.040), and there was no significant statistical difference in cardiopulmonary bypass time (*P*=0.560) or cross-clamping time (*P*=0.723). MAG group had a predominant use of BITA (62.86%), followed by radial graft associated with the use of left internal thoracic artery (28.57%) and combination of the three grafts (8.57%), with 28.57% not using venous grafts.

**Table 3:** Propensity score matching intraoperative variables.

Variable	SAGs		MAGs		*P*-value
	(n=35)		(n=35)		
	n	%	n	%	
CPB	34	97.14%	35	100%	1
CPB time (min.), mean ± SD	79.59	33.66	74.40	31.83	0.560
Aortic cross-clamping time (min.), mean ± SD	61.21	± 31.21	59.20	± 28.37	0.723
Radial artery	0	0%	13	37.14%	< 0.001
Right internal thoracic artery					< 0.001
Pedicled	0	0%	10	28.57%	
Skeletonized	0	0%	15	42.86%	
Left internal thoracic artery					0.015
Pedicled	25	71.43%	14	40%	
Skeletonized	10	28.57%	21	60%	
Saphenous vein	35	100%	25	71.43%	0.002
Need for transfusion of packed red blood cells	6	17.14%	4	11.43%	0.734
Porcelain aorta	0	0%	3	8.57%	0.238
Lower temperature (°C), mean ± SD	33.28	± 1.5	33.57	± 1.85	0.694
Higher intraoperative blood glucose, mean ± SD	178.51	± 60.06	196.66	± 68.23	0.247
Lower intraoperative hematocrit, mean ± SD	26.70+B90:B91	± 5.28	27.31	± 4.8	0.485
Lower intraoperative hemoglobin, mean ± SD	9.70	± 4.84	8.98	± 1.63	0.825
Surgery duration^*^ (hours), mean ± SD	4.11	± 1.24	4.78	± 1.17	0.040
Operating room extubation	0	0%	4	11.43%	0.122

CPB=cardiopulmonary bypass; MAG=multiple arterial grafts; SAG=single arterial graft; SD=standard deviation

^*^Period between surgical incision and referral to the intensive care unit

No significant differences were found in the immediate outcomes (Table 4).

**Table 4:** Propensity score matching variables from the follow-up (up to 30 days).

Variable	SAGs		MAGs		*P*-value
	(n=35)		(n=35)		
	n	%	n	%	
Need for an IABP	2	5.71%	0	0%	0.473
Need for reintubation	0	0%	1	2.86%	1
Atrial fibrillation	5	35.71%	4	25%	0.694
Infection (thoracotomy)	2	50%	5	71.43%	0.339
Graft harvest site infection	3	75%	1	14.29%	0.088
Deep wound infection/mediastinitis	0	0%	3	42.86%	0.406
Sepsis	0	0%	2	12.50%	0.525
Superficial wound infection	3	75%	4	57.14%	1
Pleural effusion with indication for drainage	0	0%	2	12.50%	0.525
Cerebrovascular accident	1	7.14%	0	0%	0.946
Reoperation/procedure due to infectious complication	0	0%	5	71.43%	0.097
Cardiac arrest	0	0%	1	6.25%	1
Pneumonia	0	0%	2	12.50%	0.525
Peak of blood glucose 18-24 hours postoperatively, mean ± SD	169.17	± 46.98	165.76	± 32.24	0.522
Hemoglobin at hospital discharge, mean ± SD	10.24	± 1.46	11.41	± 5.96	0.959
Hematocrit at hospital discharge, mean ± SD	31.15	± 0.04	28.97	± 0.06	0.270
LVEF (%) before hospital discharge, mean ± SD	46.52	± 0.23	53.13	± 0.18	0.541
Higher postoperative creatinine (mg/dL), mean ± SD	1.46	± 0.73	1.38	± 0.55	0.966
Postoperative orotracheal intubation time (hours), mean ± SD	5.99	± 0.04	6.18	± 0.04	0.984
ICU length of stay (hours)	77.71	± 50.43	57.09	± 26.23	0.135
Postoperative hospital length of stay (days)	7.37	± 3.46	6.94	± 2.44	0.924
Readmission up to 30 days after hospital discharge	0	0%	1	2.45%	0.948

IABP=intra-aortic balloon pump; ICU=intensive care unit; LVEF=left ventricular ejection fraction; MAG=multiple arterial grafts; SAG=single arterial graft; SD=standard deviation

Regarding the mid-term follow-up (3-5 years after surgery) in Table 5, 11.43% of patients were lost to follow-up, resulting in a total of 62 patients to be analyzed, 31 in each group. There was one fatal new AMI case in the SAG group, and two cases in the MAG group, where one patient died. With regard to hospital readmissions, non-cardiac causes (77%) were more frequent than cardiac causes (23%) in both groups, and the same applies to the causes of death, 75% and 25% respectively. There were two cases of angioplasty in the MAG group and one case of redo CABG in the SAG group.

**Table 5:** Propensity score matching mid-term follow-up variables.

Variable	SAGs		MAGs		*P*-value
	(n=31)		(n=31)		
	n	%	n	%	
NYHA classification					0.664
I	15	75%	19	67.86	
II	4	20%	6	21.43%	
III	1	5%	1	3.57%	
IV	0	0%	2	7.14%	
CCS angina classification					0.168
I	18	90%	27	96%	
II	2	10%	0	0%	
III	0	0%	0	0%	
IV	0	0%	1	4%	
Hospital readmission					
Total	5	16.13%	8	25.81%	0.55
Cardiac reasons	1	3.23%	2	6.45%	0.62
Non-cardiac reasons	4	16.13%	6	19.35%	
Main cause of hospital readmission					0.55
AMI	1	3.23%	2	6.45%	
Surgical site infection	2	6.45%	0	0%	
Pleural effusion requiring thoracentesis	0	0%	1	3.23%	
Unspecified neoplasm	0	0%	1	3.23%	
Others^*^	2	6.45%	4	12.90%	
Angioplasty	0	0%	2	6.45%	-
Reoperation	1	3.23%	0	0%	-
Death					
Total	5	16.13	3	9.68	0.44
Cardiac reasons	1	3.23%	1	3.23%	0.83
Non-cardiac reasons	4	12.90%	2	6.45%	
Cause of death					0.67
AMI	1	3.23%	1	3.23%	
Unspecified respiratory failure	0	0%	1	3.23%	
Unspecified neoplasm	2	6.45%	1	3.23%	
COVID-19	1	3.23%	0	0%	
Brain aneurysm	1	3.23%	0	0%	

* Others: appendicitis, diverticulitis, complications related to poorly controlled diabetes mellitus

AMI=acute phase of myocardial infarction; CCS=Canadian Cardiovascular Society; COVID-19=coronavirus disease 2019; MAG=multiple arterial grafts; NYHA=New York Heart Association; SAG=single arterial graft

**Fig. 2:**
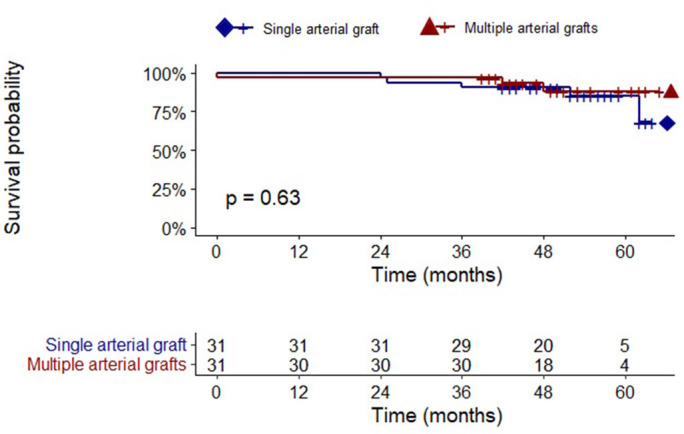
Kaplan-Meier-survival curves for death outcome by the evaluated groups.

The authors present a Kaplan-Meier survival curve (Figure 2), where is observed a superior survival probability for the MAGs group (*P*=0.63).

## DISCUSSION

This study provides evidence that the use of MAGs during CABG in the AMI does not cause harm in the short and mid-terms when compared with the use of a SAG. This comes to fill a gap in relation to the use of the best grafts during the AMI in patients operated on urgently. Approximately 5-10% of patients in the AMI require CABG^[12,13]^ and represent a challenging subgroup due to their high-risk characteristics compared to patients undergoing elective CABG.

The advantages of the use of MAGs are related to the long term^[6]^, even with a higher rate of infection, technical difficulty, and increased surgical duration^[14,15]^. The present analysis in patients in the AMI shows that the group with MAGs did not present a clinically significant difference in relation to the use of a SAG in the mid-term. Therefore, although there is a preference for not using MAGs in the AMI, this should be guided by thinking about the long-term benefits.

Taggart et al.^[16]^, in the Arterial Revascularization Trial (or ART), analyzed randomized patients regarding the impact of MAGs *vs.* SAG on CABG. In the mid-term follow-up, only the higher rate of deep sternal wound infection in the MAGs was pointed. Our study, which also included a radial graft in the MAG group in patients in the AMI, also showed no mid-term differences in relation to the clinical outcomes analyzed, with a tendency towards a higher rate of deep sternal wound infection.

In the immediate postoperative period, the SAG group had a slight increase in hours spent in the intensive care unit (77.71 ± 50.43 *vs.* 57.09 ± 26.23, *P*=0.135) and hospital stay (7.37 ± 3.46 *vs.* 6.94 ± 2.44, *P*=0.924). We believe that the potential for a reduction in the length of hospital stay in the MAG group is influenced by early postoperative ambulation due to the non-use of the saphenous vein, culminating in both clinical and psychological improvements already described in rapid recovery protocols^[17]^.

Although the groups had a similar baseline glycemic profile, a greater tendency for complications of surgical wounds was demonstrated in the MAG group, a cause already known for rejection of the multiple arterial technique by many health teams^[14,15]^. The graft choice falls short of adequate glycemic control since the rigorous management of glycemic parameters is crucial for the positive evolution of patient’s outcomes. In our analysis, there was a less rigid interoperative management of glycemic parameters in the MAGs group, where the higher blood glucose in the group reached an average of 196.66mg/dL (*P*=0.247) followed by a higher frequency of infections outcomes.

Dorman et al.^[18]^ compared controlled diabetic patients undergoing CABG with SAG and MAGs in a 30-year follow-up and demonstrated that the use of MAGs did not increase morbidity or mortality rates; Zhou et al.^[19]^ also evaluated this patient profile and showed benefits in the use of the MAGs for well-controlled diabetic patients. This supports the hypothesis that, regardless of whether the diagnosis of diabetes, what influences surgical wound infection is the perioperative glycemic control.

About hospital readmission within 30 days of discharge, one patient in the MAG group had to return due to pleural effusion requiring thoracentesis. In the mid-term follow-up, five patients in the SAG group *vs.* eight patients in the MAG needed to return to the hospital, mostly due to non-cardiac causes. In the MAG group, the need for angioplasty was observed in two cases, while in the SAG there was a reoperation and a new CABG.

The SAG group had a higher mortality, although not statistically significant, with five (16.13%) deaths *vs.* three (9.68%, *P*=0.44). The occurrence of a new myocardial infarction with fatal outcome was observed in only one case in each group, given that deaths from non-cardiac causes were more frequent. Enezate et al.^[20]^ evaluated the difference between percutaneous revascularization and CABG for AMI patients, separating SAGs and MAGs, demonstrating that there were benefits related to a reduction in the mortality rate in the MAG group, similar to our findings.

Grothusen et al.^[21]^ retrospectively studied patients who had AMI complicated by cardiogenic shock, submitted to CABG hours after the diagnosis, showing that surgical treatment can bring benefits to patients, however, the techniques of SAG *vs.* MAG were not considered. In this scenario, more studies still need to be carried out to correlate the best AMI patient care strategy, providing satisfactory clinical evolution and longevity.

### Limitations

The decision on which graft to use did not follow a standardization in this study, then, to reduce this bias PSM was used in order to pair the two groups of patients who underwent CABG (SAG and MAG group), in order to assess its impacts on patient evolution. Despite the promising results obtained, it is important to highlight that the sample size used in this study limits the generalization of findings to the general population, the results need to be validated in future research, preferably in randomized studies, considered gold standard method.

The surgeon's experience is a crucial point in choosing the technique used^[22]^. In our analysis, the volume of MAGs procedures per team in each of the institutions was not considered, therefore, a more detailed analysis for this purpose can help to elucidate and better understand the results obtained.

According to the literature, we believe that the MAGs CABG is superior to the SAG due to the long-term graft patency^[4]^. However, it is necessary to consider the clinical status of the patient and the technical experience of the surgeon and the team, to adequately prepare the patient, focusing on perioperative glycemic control and optimization of surgical times.

## CONCLUSION

In this analysis, the use of MAGs, even in the AMI, did not bring disadvantages in the shortand mid-term follow-ups compared to patients with a SAG. Therefore, we encourage the use of MAGs given the long-term benefits for patients.

## Data Availability

The REPLICCAR II database used to support the findings of this study have not been made available due to ethical restrictions: patients did not consent to their data being publicly shared. De-identified data can be made available to qualified researchers under their responsibility and assuming the penalties if public disclosure of the data. Data requests should be sent to Renata do Val, Director of the Scientific Committee, Ethics Committee of the Instituto do Coração-Universidade de São Paulo (renata.doval@incor.usp.br http://www.incor.usp.br/sites/incor2013/index.php/equipe/16-pesquisa/comissao-cientifica/158-fale-conosco [1]) or Prof. Dr. Alfredo José Mansur, Coordinator, CAPPesq (cappesq.adm@hc.fm.usp.br http://www.hc.fm.usp.br/index.php?option=com_content&view=article&id=243:comissao-de-etica-para-analise-de-projetos-de-pesquisa-do-hcfmusp&catid=23&Itemid=229 [2]).

## Abbreviations, Acronyms & Symbols

ACSD: = Adult Cardiac Surgery Database
AMI: = Acute phase of myocardial infarction
BITA: = Bilateral internal thoracic artery
CABG: = Coronary artery bypass grafting
CAPPesq: = Comissão de Ética para Análise de Projetos de Pesquisa
CCS: = Canadian Cardiovascular Society
COVID-19: = Coronavirus disease 2019
CPB: = Cardiopulmonary bypass
IABP: = Intra-aortic balloon pump
ICU: = Intensive care unit
LVEF: = Left ventricular ejection fraction
MAGs: = Multiple arterial grafts
NYHA: = New York Heart Association
PRA-MD: = Prasugrel maintenance dose
PSM: = Propensity score matching
REPLICCAR: = Registro Paulista de Cirurgia Cardiovascular
SAG: = Single arterial graft
SD: = Standard deviation
SMD: = Standardized mean difference
STEMI: = ST-elevation myocardial infarction
STS: = Society of Thoracic Surgeons

## References

[1] Locker C, Schaff HV, Daly RC, Bell MR, Frye RL, Stulak JM (2017). Multiarterial grafts improve the rate of early major adverse
cardiac and cerebrovascular events in patients undergoing coronary
revascularization: analysis of 12 615 patients with multivessel
disease. Eur J Cardiothorac Surg.

[2] Schwann TA, Habib RH, Wallace A, Shahian D, Gaudino M, Kurlansky P (2019). Bilateral internal thoracic artery versus radial artery
multi-arterial bypass grafting: a report from the STS
database†. Eur J Cardiothorac Surg.

[3] Gaudino M, Bakaeen FG, Benedetto U, Di Franco A, Fremes S, Glineur D (2019). Arterial grafts for coronary bypass: a critical review after the
publication of ART and RADIAL. Circulation.

[4] Vallely MP, Ramponi F, Seco M, Royse A. (2021). Multiarterial grafting: why is it so hard to convince the masses
of the benefits?. J Thorac Cardiovasc Surg.

[5] Muneretto C, Negri A, Manfredi J, Terrini A, Rodella G, Elqarra S (2003). Safety and usefulness of composite grafts for total arterial
myocardial revascularization: a prospective randomized
evaluation. J Thorac Cardiovasc Surg.

[6] Tatoulis J, Buxton BF, Fuller JA. (2004). Patencies of 2127 arterial to coronary conduits over 15
years. Ann Thorac Surg.

[7] Verma S, Szmitko PE, Weisel RD, Bonneau D, Latter D, Errett L (2004). Should radial arteries be used routinely for coronary artery
bypass grafting?. Circulation.

[8] Samadashvili Z, Sundt TM, Wechsler A, Chikwe J, Adams DH, Smith CR (2019). Multiple versus single arterial coronary bypass graft surgery for
multivessel disease. J Am Coll Cardiol.

[9] Thygesen K, Alpert JS, Jaffe AS, Chaitman BR, Bax JJ, Morrow DA (2018). Fourth universal definition of myocardial infarction
(2018). J Am Coll Cardiol.

[10] Lawton JS, Tamis-Holland JE, Bangalore S, Bates ER, Beckie TM, Bischoff JM (2022). 2021 ACC/AHA/SCAI guideline for coronary artery
revascularization: executive summary: a report of the American college of
cardiology/American heart association joint committee on clinical practice
guidelines. Circulation.

[11] Society of Thoracic Surgeons (2011). STS Adult Cardiac Database Data Specifications Version 2.9.

[12] Ranasinghe I, Alprandi-Costa B, Chow V, Elliott JM, Waites J, Counsell JT (2011). Risk stratification in the setting of non-ST elevation acute
coronary syndromes 1999-2007. Am J Cardiol.

[13] Fukui T, Tabata M, Morita S, Takanashi S. (2013). Early and long-term outcomes of coronary artery bypass grafting
in patients with acute coronary syndrome versus stable angina
pectoris. J Thorac Cardiovasc Surg.

[14] Schwann TA, Habib RH, Wallace A, Shahian DM, O'Brien S, Jacobs JP (2018). Operative outcomes of multiple-arterial versus single-arterial
coronary bypass grafting. Ann Thorac Surg.

[15] Taggart DP, Altman DG, Gray AM, Lees B, Gerry S, Benedetto U (2016). Randomized trial of bilateral versus single
internal-thoracic-artery grafts. N Engl J Med.

[16] Mejia OAV, Borgomoni GB, Lasta N, Okada MY, Gomes MSB, Foz MLNN (2021). Safe and effective protocol for discharge 3 days after cardiac
surgery. Sci Rep.

[17] Dorman MJ, Kurlansky PA, Traad EA, Galbut DL, Zucker M, Ebra G. (2012). Bilateral internal mammary artery grafting enhances survival in
diabetic patients: a 30-year follow-up of propensity score-matched
cohorts. Circulation.

[18] Zhou P, Zhu P, Nie Z, Zheng S. (2019). Is the era of bilateral internal thoracic artery grafting coming
for diabetic patients? An updated meta-analysis. J Thorac Cardiovasc Surg.

[19] Enezate T, Gifft K, Chen C, Omran J, Eniezat M, Reardon M. (2021). Percutaneous versus surgical revascularization for acute
myocardial infarction. Cardiovasc Revasc Med.

[20] Grothusen C, Friedrich C, Ulbricht U, Meinert J, Attmann T, Huenges K (2022). Coronary artery bypass grafting in patients with acute myocardial
infarction and cardiogenic shock. Rev Cardiovasc Med.

[21] Taggart DP. (2019). The role of multiple arterial grafts in CABG: all roads lead to
ROMA. J Am Coll Cardiol.

